# Association between six anthropometric indices and incident arthritis: prospective findings from the English Longitudinal Study of Ageing

**DOI:** 10.3389/fnut.2026.1824752

**Published:** 2026-05-25

**Authors:** Yanqi Du, Ying Wang, Zhen Huang, Yaobo Liu

**Affiliations:** 1 Department of Trauma Center, Jinan Central Hospital Affiliated to Shandong First Medical University, Jinan, Shandong, China; 2 Department of Internal Medicine, Jinan Lixia District People's Hospital, Jinan, Shandong, China; 3 Department of Joint Surgery, Minda Hospital of Hubei Minzu University, Enshi, Hubei, China

**Keywords:** anthropometric indices, arthritis, body roundness index, ELSA, obesity, prospective cohort

## Abstract

**Background:**

Arthritis, predominantly osteoarthritis in older adults, imposes a substantial burden on quality of life and healthcare systems. Obesity is a well-established modifiable risk factor; however, traditional body mass index (BMI) has limitations in capturing visceral adiposity and central obesity. Novel anthropometric indices, including the Body Roundness Index (BRI), A Body Shape Index (ABSI), weight-adjusted waist index (WWI), waist-to-height ratio (WHtR), waist circumference (WC), and BMI, may offer additional insights. This study examined their associations with incident arthritis in a prospective cohort of elderly individuals.

**Methods:**

We analyzed data from 4,112 participants without arthritis from the English Longitudinal Study of Ageing (ELSA) Wave 2 (2004–2005) and followed them until Wave 9 (2018–2019). Height, weight, and WC were objectively measured by trained nurses. Anthropometric indices were calculated using standard formulas and standardized to per-standard deviation (SD) increments and tertiles (Q1: lowest; Q2: medium; Q3: highest). Incident arthritis was defined as the first self-reported physician-diagnosed arthritis during the follow-up period. Cox proportional hazards models were used to estimate hazard ratios (HRs) and 95% confidence intervals (CIs), after adjusting for age, sex, ethnicity, education, marital status, smoking status, drinking status, hypertension, diabetes, stroke, high cholesterol levels, congestive heart failure, psychological problems, the Control, Autonomy, Self-realization and Pleasure-19 (CASP-19) scale scores, and physical activity. Subgroup analyses and interaction tests were performed. Kaplan–Meier curves and restricted cubic splines (RCS) were used to assess cumulative incidence and dose–response relationships.

**Results:**

During a median follow-up of 14.2 years, 944 incident arthritis cases occurred (23%). After fully adjusting for all covariates, per-SD increases in anthropometric indices such as the BRI (HR: 1.17, 95% CI: 1.10–1.25), WHtR (HR: 1.18, 95% CI: 1.10–1.27), WC (HR: 1.21, 95% CI: 1.13–1.31), BMI (HR: 1.20, 95% CI: 1.12–1.28), and WWI (HR: 1.09, 95% CI: 1.01–1.18) were significantly associated with a higher incidence of arthritis. However, the ABSI showed no association (HR: 1.01, 95% CI: 0.92–1.10). RCS analyses confirmed that there are predominantly linear positive dose–response relationships for the BRI, WHtR, WC, and BMI (all *p* values for non-linearity > 0.05). Tertile analyses revealed clear dose–response trends for the majority of indices, with participants in the high tertile having significantly greater risk of incident arthritis compared to those in the low tertile.

**Conclusion:**

Anthropometric indices, excluding the ABSI, were associated with increased incident arthritis risk in middle-aged and older adults.

## Introduction

Arthritis affects an estimated 53.2 million adults in the United States and remains one of the leading causes of disability worldwide, with prevalence increasing sharply after age 50 (1). Similar patterns are observed in the United Kingdom and other high-income countries, where arthritis is particularly common among older adults, women, and individuals with lower socioeconomic status (2). While rheumatoid arthritis involves autoimmune mechanisms, the vast majority of arthritis cases in older populations are attributable to osteoarthritis, although self-reported diagnoses often encompass a range of arthritic conditions (3).

Obesity, traditionally defined by body mass index (BMI) ≥ 30 kg/m^2^, is a well-established modifiable risk factor for incident arthritis, particularly in weight-bearing joints (4–6). Large-scale meta-analyses of prospective studies consistently demonstrate a clear dose–response relationship: overweight (BMI 25–29.9 kg/m^2^) and obesity are associated with an markedly elevated risk, particularly in weight-bearing joints such as the knee, with each 5 kg/m^2^ increase in BMI linked to approximately a 35% higher risk (4, 5).

Mechanistically, obesity may contribute through both biomechanical overload on weight-bearing joints and systemic metabolic effects, including low-grade inflammation driven by adipose tissues (3, 7).

Despite its utility, BMI has significant limitations. It does not distinguish fat distribution, muscle mass, and visceral adipose tissue (VAT) accumulation, which is more strongly linked to metabolic and inflammatory dysregulation than subcutaneous fat (8). The Body Roundness Index (BRI) models the body as an ellipse using waist circumference (WC) and height, providing a proxy for VAT and roundness (8). A Body Shape Index (ABSI) isolates abdominal shape independent of BMI and height, aiming to predict mortality and metabolic risks beyond general obesity (9). The weight-adjusted waist index (WWI) normalizes WC for body weight, reflecting fat relative to muscle mass (10). The waist-to-height ratio (WHtR) and WC directly assess central adiposity, which are often useful in cardiometabolic prediction (11).

Cross-sectional studies, predominantly based on data from the National Health and Nutrition Examination Survey (NHANES), have consistently linked higher levels of central and novel anthropometric indices, such as BRI, WHtR, WC, and in some cases ABSI, to increased prevalence of arthritis (12–14).

For instance, the elevated BRI is positively associated with arthritis prevalence, often non-linearly, and may better capture VAT-related inflammation (12, 15). ABSI shows mixed results, with positive associations in some subgroups (men and younger adults) but weaker overall (13). Longitudinal evidence remains sparse, particularly in European elderly populations, where body composition changes may alter index performance.

The English Longitudinal Study of Ageing (ELSA), a nationally representative cohort of English adults aged ≥ 50 years, offers an ideal setting: Wave 2 (2004–2005) included nurse-measured anthropometrics (height, weight, and WC), and repeated waves captured incident self-reported physician-diagnosed arthritis up to Wave 9 (2018–2019) (16).

Previous ELSA analyses have linked psychosocial factors, inflammation, and multimorbidity to arthritis incidence (17). None has systematically compared these six indices (BRI, ABSI, WWI, WHtR, WC, and BMI) for prospective associations with incident arthritis.

We focused on the ELSA because it is the largest nationally representative cohort in England with objectively nurse-measured anthropometrics and repeated arthritis assessments. Although other UK cohorts (such as Healthy Ageing in Scotland) exist, data access was limited to the ELSA for the present analysis.

This study aimed to examine the prospective associations between six anthropometric indices (BRI, ABSI, WWI, WHtR, WC, and BMI) and incident self-reported physician-diagnosed arthritis in a large cohort of middle-aged and older English adults.

## Methods

### Study population and design

The ELSA is an ongoing multidisciplinary panel study of community-dwelling adults aged ≥ 50 years in England. Details of the sampling, study design, and data collection have been published previously (16).

The present analysis used Wave 2 (2004–2005) as the baseline because it was the first wave with comprehensive nurse-measured anthropometric data. Participants were followed biennially until Wave 9 (2018–2019), providing up to 14.2 years of follow-up.

**Figure 1:**
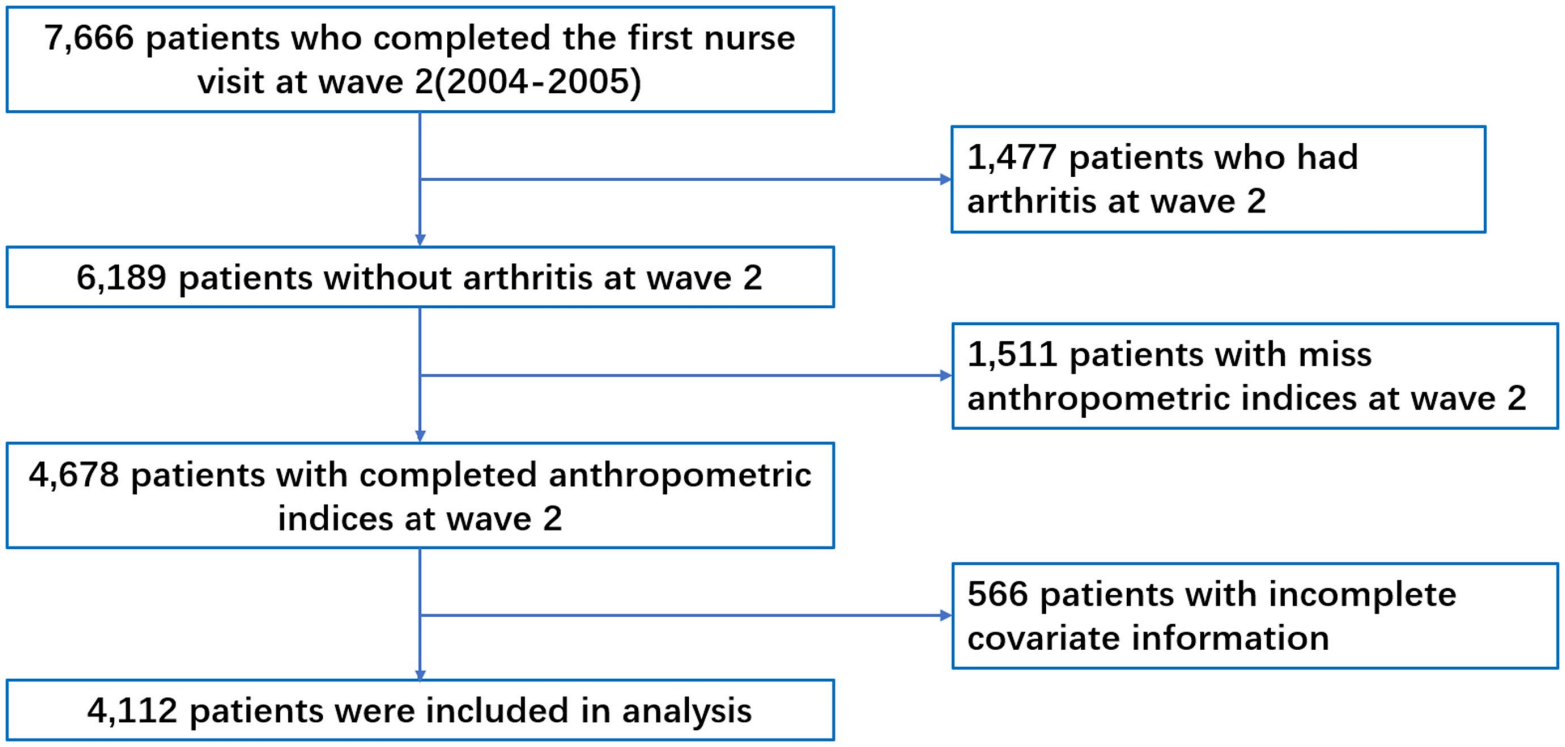
Flow chart of the study participants.

Participants with prevalent physician-diagnosed arthritis at Wave 2, missing anthropometric data, or incomplete information on any covariate (age, sex, ethnicity, education, marital status, smoking status, drinking status, hypertension, diabetes, stroke, high cholesterol, congestive heart failure, psychological problems, CASP-19, or moderate-to-vigorous physical activity) were excluded, resulting in a final analytic sample of 4,112 arthritis-free participants using a complete-case approach (see Figure 1).

### Anthropometric measurements and indices

All anthropometric measurements were performed by trained study nurses during the Wave 2 nurse visit, following standardized protocols to minimize measurement error and ensure reproducibility.

Standing height was measured to the nearest 0.1 cm using a portable stadiometer. Body weight was recorded to the nearest 0.1 kg using calibrated Tanita digital scales, with participants wearing light clothing and no shoes.

WC was measured to the nearest 0.1 cm at the midpoint between the lowest rib margin and the iliac crest, using a non-stretchable tape measure, with participants standing and breathing normally (16).

All measurements were taken twice, and the average of the two readings was used for analysis (or the single valid measurement if only one was available).

These objective measurements were used to compute the following anthropometric indices (12, 15), with all inputs expressed in consistent units (height in meters for BMI, centimeters for WC and height in other indices, weight in kilograms):

$$\mathrm{BMI}=\text{weight}\;(\mathrm{kg})/{\left[\text{height}\;(m)\right]}^{2}$$

WC=measured waist circumference(cm)

WHtR=WC(cm)/height(cm)

$$\mathrm{BRI}=364.2–365.5\times \surd [1-(\begin{array}{l} {\left(\mathrm{WC}(\mathrm{cm})/(2\pi )\right)}^{2}/\\ {\left(0.5\times \text{height}(\mathrm{cm})\right)}^{2} \end{array})]$$

ABSI=WC/(BMI^(2/3)×height^0.5)

WWI=WC(cm)/√weight(kg)

### Outcome

Incident arthritis was ascertained through self-reported physician-diagnosed arthritis at follow-up waves (Waves 3 through 9). At each wave, the participants were asked: “Has a doctor ever told you that you have arthritis (including osteoarthritis or rheumatism)?”

Incident cases were defined as the first positive response among arthritis-free participants at baseline (Wave 2) (18, 19).

This broad definition does not distinguish between osteoarthritis and other forms of arthritis (e.g., rheumatoid arthritis) and does not specify affected joint sites.

### Covariates

A set of baseline covariates was selected *a priori* based on established risk factors for arthritis and potential confounders of the adiposity–arthritis relationship. These covariates were assessed at Wave 2 and included the following:

Sociodemographic factors: age, sex (male/female), ethnicity (White vs. other), education level (categorized as below high school, high school, college or above, or other), and marital status (married or partnered, never married, separated/divorced/widowed).

*Lifestyle factors*: smoking status (current, ever, never) and drinking status (ever drinker vs. never drinker).

Medical history and comorbidities: self-reported physician-diagnosed hypertension (or current antihypertensive medication use), diabetes, stroke, high cholesterol, congestive heart failure, and psychological problems (self-reported physician diagnosis or relevant medication use).

*Psychosocial well-being*: CASP-19 quality-of-life score (continuous; higher scores indicate better quality of life), a validated 19-item scale measuring control, autonomy, self-realization, and pleasure (20).

Moderate-to-vigorous physical activity was defined as 1 if the participant reported engaging in moderate or vigorous physical activity more than once a week and 0 otherwise (21).

### Statistical analysis

All analyses were performed using a complete-case approach (final analytic sample: *n* = 4,112).

Baseline characteristics were summarized as means with standard deviations (SDs) for continuous variables and frequencies with percentages (*n*, %) for categorical variables.

To ensure consistency and comparability across the anthropometric indices, all indices were standardized using the SD derived from the analytic sample, with hazard ratios (HRs) expressed as the change in arthritis risk per 1-SD increase.

Cumulative incidence of arthritis by tertiles of each anthropometric index was visualized using Kaplan–Meier curves, with differences across tertiles assessed using the log-rank test.

To explore the shape of the dose–response relationship between each standardized anthropometric index and incident arthritis, restricted cubic spline (RCS) models with four knots were fitted within the fully adjusted model (age, sex, ethnicity, education, marital status, smoking status, drinking status, hypertension, diabetes, stroke, high cholesterol, congestive heart failure, psychological problems, CASP-19, and physical activity).

Non-linearity was formally tested by comparing the spline model with the linear model using likelihood ratio tests (*P* for non-linearity).

Cox proportional hazards regression was then used to estimate HRs and 95% confidence intervals (CIs) for the associations between each anthropometric index and incident arthritis.

Two modeling approaches were applied:

1. Crude (unadjusted) models.
2. Multivariable-adjusted models controlling for all prespecified baseline covariates: age, sex, ethnicity, education, marital status, smoking status, drinking status, hypertension, diabetes, stroke, high cholesterol, congestive heart failure, psychological problems, CASP-19, and physical activity.

Subgroup analyses were performed to examine potential effect modification by prespecified factors: age (<65 vs. ≥65 years), sex, ethnicity, hypertension (yes/no), diabetes (yes/no), and smoking status (current/ever/never).

Stratified HRs were estimated within subgroups, and formal interaction tests were conducted using likelihood ratio tests comparing models with and without the interaction term (*P* for interaction).

All analyses were conducted using R version 4.4.3. A two-sided *p*-value < 0.05 was considered statistically significant.

### Baseline characteristics

The analytic cohort comprised 4,112 participants free of arthritis at baseline (ELSA Wave 2), with a mean age of 64.90 ± 8.95 years; 49.8% were women, and 98.9% were of White ethnicity (Table 1).

**Table 1:** Baseline characteristics of the study population (*n* = 4,112).

Variable	Overall
Number	4,112
Age, year [mean (SD)]	64.90 (8.95)
Sex, *n* (%)	
Female	2,048 (49.8)
Male	2,064 (50.2)
Ethnicity, *n* (%)	
White	4,065 (98.9)
Others	47 (1.1)
Education, *n* (%)	
Below high school	1,490 (36.2)
College or above	1,482 (36.0)
High school	783 (19.0)
Other	357 (8.7)
Marital status, *n* (%)	
Married or partnered	3,069 (74.6)
Never married	173 (4.2)
Separated/divorced/widowed	870 (21.2)
Smoking status, *n* (%)	
Current smokers	575 (14.0)
Ever smokers	1,977 (48.1)
Never smokers	1,560 (37.9)
Drinking status, *n* (%)	
Ever drinkers	3,774 (91.8)
Never drinkers	338 (8.2)
Hypertension, *n* (%)	
No	2,620 (63.7)
Yes	1,492 (36.3)
Diabetes, *n* (%)	
No	3,840 (93.4)
Yes	272 (6.6)
Psych problems, *n* (%)	
No	3,821 (92.9)
Yes	291 (7.1)
Stroke, *n* (%)	
No	3,987 (97.0)
Yes	125 (3.0)
High cholesterol, *n* (%)	
No	3,432 (83.5)
Yes	679 (16.5)
Congestive heart failure, *n* (%)	
No	4,097 (99.6)
Yes	15 (0.4)
CASP-19 (mean (SD))	44.42 (7.86)
Moderate-to-vigorous physical activity, *n* (%)	
No	3,200 (77.8)
Yes	912 (22.2)

Data are from ELSA Wave 2 baseline (2004–2005). Continuous variables are presented as mean (SD) and categorical variables as *n* (%).

SD, standard deviation; CASP-19, Control, Autonomy, Self-realization and Pleasure-19 quality-of-life scale.

Education distribution showed 36.2% of participants had an education below high school level, whereas 36.0% had a college education or above. Most participants were married or partnered (74.6%).

Smoking status included 14.0% current smokers and 48.1% ever smokers, whereas 91.8% were ever drinkers.

Comorbidities were prevalent, including hypertension in 36.3%, diabetes in 6.6%, psychological problems in 7.1%, stroke in 3.0%, high cholesterol in 16.5%, and congestive heart failure in 0.4%.

The mean CASP-19 quality-of-life score was 44.42 ± 7.86.

Across tertiles of BRI, WC, WWI, WHtR, BMI, and ABSI, clear gradients were observed for age, sex, education, marital status, smoking status, hypertension, and diabetes (mostly *p* < 0.001). Participants in the highest tertile were generally older, more likely to be female, less educated, and had a higher prevalence of hypertension and diabetes, as well as lower levels of moderate-to-vigorous physical activity.

CASP-19 quality-of-life scores were consistently lower in the highest tertile of all six indices (*p* < 0.01), whereas psychological problems showed mostly non-significant differences across groups (*p* > 0.05).

WC, WHtR, BRI, ABSI, and WWI showed strong associations with metabolic comorbidities (Supplementary Tables S1–S6).

### Association between six anthropometric indices and incident arthritis

**Figure 2:**
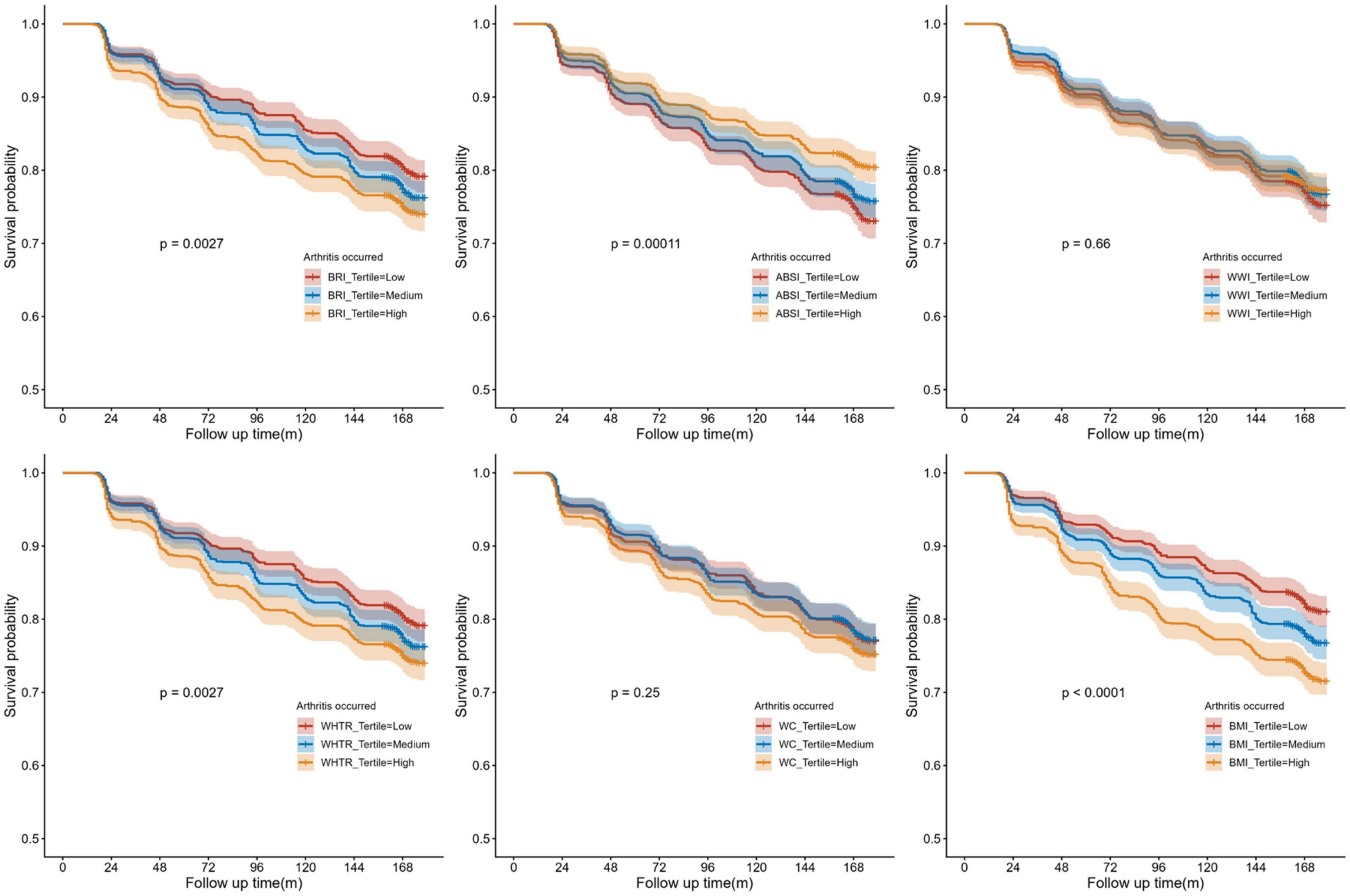
Kaplan–Meier curves of cumulative incidence of arthritis according to tertiles of each anthropometric index.

During a median follow-up of 14.2 years, Kaplan–Meier survival curves showed clear separation across tertiles for the majority of indices (log-rank *p* < 0.01 for BRI, ABSI, WHtR, and BMI), with the highest tertiles consistently exhibiting the steepest cumulative incidence (Figure 2).

**Figure 3:**
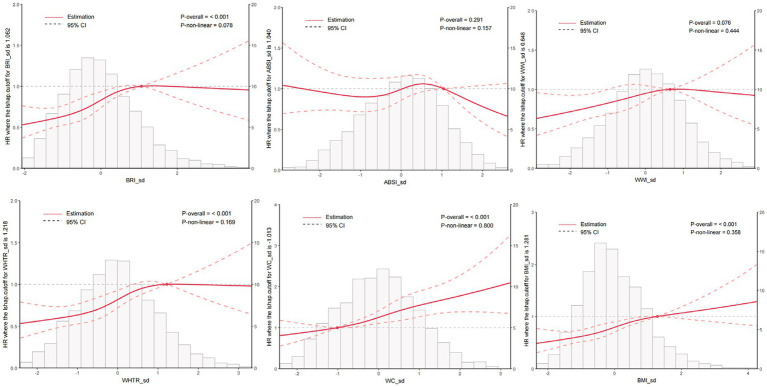
Restricted cubic spline analysis of the dose–response relationship between each standardized anthropometric index and incident arthritis risk (adjusted model).

RCS analyses confirmed predominantly linear positive dose–response relationships for BRI, WHtR, WC, and BMI (all *P* for non-linearity > 0.05) (Figure 3).

In unadjusted models, per-1-SD increases in BRI (HR: 1.13, 95% CI: 1.06–1.20), WHtR (HR: 1.13, 95% CI: 1.07–1.21), WC (HR: 1.07, 95% CI: 1.00–1.14), ABSI (HR: 0.86, 95% CI: 0.81–0.91) and BMI (HR: 1.22, 95% CI: 1.15–1.30) were positively associated with incident arthritis, whereas WWI (HR: 0.98, 95% CI: 0.92–1.05) showed no significant associations.

After full multivariable adjustment, positive associations were found for BRI (HR: 1.17, 95% CI: 1.10–1.25, *p* < 0.001), WHtR (HR: 1.18, 95% CI: 1.10–1.27), WC (HR: 1.21, 95% CI: 1.13–1.31), BMI (HR: 1.20, 95% CI: 1.12–1.28), and WWI (HR: 1.09, 95% CI: 1.01–1.18), whereas ABSI was not significantly associated with incident arthritis (HR: 1.01, 95% CI: 0.92–1.10, *p* = 0.918).

High levels of BRI (Q3 HR: 1.49, 95% CI: 1.25–1.77), WWI (Q3 HR: 1.22, 95% CI: 1.02–1.46), WHtR (Q3 HR: 1.49, 95% CI: 1.25–1.77), WC (Q3 HR: 1.51, 95% CI: 1.25–1.82), BMI (Q3 HR: 1.59, 95% CI: 1.34–1.89) were associated with significantly elevated incident arthritis risk compared with the low tertile group.

However, the high ABSI group showed no significant difference in incident arthritis compared with the low tertile group (Q3 HR: 1.09, 95% CI: 0.88–1.34, *p* = 0.441) (Table 2).

**Table 2:** Associations between anthropometric indices (per SD and by tertiles) and incident arthritis.

Value	Event counts	Unadjusted HR	*p* value	Adjusted HR	*p* value
BRI_sd	944/4,112 (23%)	1.13 (1.06–1.20)	**<0.001**	1.17 (1.10–1.25)	**<0.001**
Q1	277/1,371 (20.2%)	Reference		Reference	
Q2	317/1,371 (23.1%)	1.16 (0.99–1.37)	0.068	1.31 (1.11–1.56)	**0.002**
Q3	350/1,370 (25.5%)	1.32 (1.13–1.54)	**<0.001**	1.49 (1.25–1.77)	**<0.001**
ABSI_sd	944/4,112 (23%)	0.86 (0.81–0.91)	**<0.001**	1.01 (0.92–1.10)	0.918
Q1	356/1,371 (26.0%)	Reference		Reference	
Q2	324/1,371 (23.6%)	0.89 (0.77–1.04)	0.146	1.12 (0.94–1.32)	0.213
Q3	264/1,370 (19.3%)	0.71 (0.60–0.83)	**<0.001**	1.09 (0.88–1.34)	0.441
WWI_sd	944/4,112 (23%)	0.98 (0.92–1.05)	0.538	1.09 (1.01–1.18)	**0.020**
Q1	326/1,371 (23.8%)	Reference		Reference	
Q2	311/1,371 (22.7%)	0.94 (0.81–1.10)	0.450	1.14 (0.96–1.35)	0.130
Q3	307/1,370 (22.4%)	0.94 (0.80–1.10)	0.410	1.22 (1.02–1.46)	**0.034**
WHtR_sd	944/4,112 (23%)	1.13 (1.07–1.21)	<0.001	1.18 (1.10–1.27)	**<0.001**
Q1	277/1,371 (20.2%)	Reference		Reference	
Q2	317/1,371 (23.1%)	1.16 (0.99–1.37)	0.068	1.31 (1.11–1.56)	**0.002**
Q3	350/1,370 (25.5%)	1.32 (1.13–1.54)	**<0.001**	1.49 (1.25–1.77)	**<0.001**
WC_sd	944/4,112 (23%)	1.07 (1.00–1.14)	**0.048**	1.21 (1.13–1.31)	**<0.001**
Q1	306/1,371 (22.3%)	Reference		Reference	
Q2	304/1,371 (22.2%)	0.99 (0.85–1.16)	0.908	1.21 (1.02–1.44)	**0.031**
Q3	334/1,370 (24.4%)	1.11 (0.95–1.30)	0.173	1.51 (1.25–1.82)	**<0.001**
BMI_sd	944/4,112 (23%)	1.22 (1.15–1.30)	**<0.001**	1.20 (1.12–1.28)	**<0.001**
Q1	353/1,371 (18.5%)	Reference		Reference	
Q2	310/1,371 (22.6%)	1.26 (1.07–1.49)	**0.007**	1.32 (1.11–1.57)	**0.002**
Q3	381/1,370 (27.8%)	1.62 (1.38–1.90)	**<0.001**	1.59 (1.34–1.89)	**<0.001**

HR, hazard ratio; CI, confidence interval; SD, standard deviation; Q1, low tertiles; Q2, middle tertiles; Q3, high tertiles; BRI, body roundness index; WC, waist circumference; WWI, weight-adjusted waist index; WHtR, waist-to-height ratio; BMI, body mass index; ABSI, a body shape index.

HRs and 95% CIs were estimated using Cox proportional hazards models. Crude models were unadjusted. Adjusted models included age, sex, ethnicity, education, marital status, smoking status, drinking status, hypertension, diabetes, stroke, high cholesterol, congestive heart failure, psychological problems, CASP-19, and physical activity. All *p* values were two-sided, and Bold values denote statistically significant results at *p* < 0.05.

### Subgroup and interaction analyses

Subgroup analyses demonstrated generally consistent per-SD associations across strata of age, sex, ethnicity, hypertension, diabetes, and smoking status.

Significant effect modification by hypertension was observed, with stronger associations among participants with hypertension than among normotensive participants for WHtR (HR: 1.27 vs. 1.12; *p* = 0.041), WC (HR: 1.32 vs. 1.14; *p* = 0.013), and BMI (HR: 1.27 vs. 1.13; *p* = 0.013) (Tables 3–8).

**Table 3:** Subgroup analysis using BRI_sd.

Variable	Count	HR (95% CI)	*p* value	*P* for interaction
Overall	944/4,112 (23%)	1.17 (1.10–1.25)	**<0.001**	
Age_group				0.771
<65	540/2,195 (24.6%)	1.16 (1.06–1.26)	**0.001**	
≥65	404/1,917 (21.1%)	1.18(1.06–1.32)	**0.002**	
Sex				0.245
Female	370/2,048 (18.1%)	1.22 (1.09–1.37)	**0.001**	
Male	574/2,064 (27.8%)	1.15 (1.05–1.25)	**0.002**	
Ethnicity				0.545
White	932/4,065 (22.9%)	1.17 (1.09–1.25)	**<0.001**	
Other	12/47 (25.5%)	0.89 (0.56–1.41)	0.610	
Hypertension				0.057
No	584/2,620 (22.3%)	1.11(1.01–1.22)	**0.028**	
Yes	360/1,492 (24.1%)	1.24 (1.12–1.36)	**<0.001**	
Diabetes				0.722
No	888/3,840 (23.1%)	1.17 (1.10–1.26)	**<0.001**	
Yes	56/272 (20.6%)	1.20 (0.87–1.66)	0.270	
Smoking status				0.406
Current smokers	116/575 (20.2%)	1.07 (0.88–1.30)	0.486	
Ever smokers	457/1,977 (23.1%)	1.24 (1.12–1.36)	**<0.001**	
Never smokers	371/1,560 (23.8%)	1.14 (1.03–1.27)	**0.016**	

HR, hazard ratio; CI, confidence interval; SD, standard deviation; BRI, body roundness index; P for interaction, *p* value for interaction from likelihood ratio test (models with vs. without interaction term).

HRs per 1-SD increase were estimated using fully adjusted Cox proportional hazards models, stratified by subgroup. Count indicates events/total (%). Full adjustment covariates were the same as those used in Table 2. All *p* values were two-sided, and Bold values denote statistically significant results at *p* < 0.05.

**Table 4:** Subgroup analysis using ABSI_sd.

Variable	Count	HR (95% CI)	*p* value	*P* for interaction
Overall	944/4,112 (23%)	1.01 (0.92–1.10)	0.918	
Age_group				0.285
<65	540/2,195 (24.6%)	1.00 (0.89–1.12)	0.987	
≥65	404/1,917 (21.1%)	1.01 (0.88–1.15)	0.920	
Sex				0.448
Female	370/2,048 (18.1%)	0.98 (0.84–1.15)	0.838	
Male	574/2,064 (27.8%)	1.02 (0.92–1.13)	0.753	
Ethnicity				0.549
White	932/4,065 (22.9%)	1.01 (0.92–1.10)	0.865	
Other	12/47 (25.5%)	7.06 (3.73–13.37)	**<0.001**	
Hypertension				0.969
No	584/2,620 (22.3%)	1.00 (0.89–1.11)	0.967	
Yes	360/1,492 (24.1%)	1.01 (0.88–1.17)	0.872	
Diabetes				0.573
No	888/3,840 (23.1%)	1.00 (0.92–1.10)	0.921	
Yes	56/272 (20.6%)	0.93 (0.62–1.41)	0.742	
Smoking status				0.109
Current smokers	116/575 (20.2%)	0.80 (0.63–1.02)	0.076	
Ever smokers	457/1,977 (23.1%)	1.00 (0.88–1.14)	0.964	
Never smokers	371/1,560 (23.8%)	1.09 (0.95–1.25)	0.217	

HR, hazard ratio; CI, confidence interval; SD, standard deviation; ABSI, a body shape index; P for interaction, *p* value for interaction from likelihood ratio test (models with vs. without interaction term).

HRs per 1-SD increase were estimated using fully adjusted Cox proportional hazards models, stratified by subgroup. Count indicates events/total (%). Full adjustment covariates were the same as those used in Table 2. All *p* values were two-sided, and Bold values denote statistically significant results at *p* < 0.05. Missing data were handled by complete-case analysis.

**Table 5:** Subgroup analysis using WWI_sd.

Variable	Count	HR (95% CI)	*p* value	*P* for interaction
Overall	944/4,112 (23%)	1.09 (1.01–1.18)	**0.020**	
Age_group				0.292
<65	540/2,195 (24.6%)	1.09 (0.99–1.21)	0.083	
≥65	404/1,917 (21.1%)	1.09 (0.97–1.23)	0.140	
Sex				0.684
Female	370/2,048 (18.1%)	1.13 (0.99–1.30)	0.069	
Male	574/2,064 (27.8%)	1.07 (0.98–1.18)	0.128	
Ethnicity				0.915
White	932/4,065 (22.9%)	1.09 (1.02–1.18)	**0.019**	
Other	12/47 (25.5%)	1.50 (0.71–3.19)	0.290	
Hypertension				0.566
No	584/2,620 (22.3%)	1.06 (0.96–1.17)	0.234	
Yes	360/1,492 (24.1%)	1.14 (1.01–1.29)	**0.037**	
Diabetes				0.434
No	888/3,840 (23.1%)	1.10 (1.02–1.18)	**0.019**	
Yes	56/272 (20.6%)	1.00 (0.69–1.45)	0.998	
Smoking status				0.204
Current smokers	116/575 (20.2%)	0.92 (0.74–1.14)	0.428	
Ever smokers	457/1,977 (23.1%)	1.13 (1.01–1.27)	**0.027**	
Never smokers	371/1,560 (23.8%)	1.12 (1.00–1.26)	0.054	

HR, hazard ratio; CI, confidence interval; SD, standard deviation; WWI, weight-adjusted waist index; P for interaction, *p* value for interaction from likelihood ratio test (models with vs. without interaction term).

HRs per 1-SD increase were estimated using fully adjusted Cox models, stratified by subgroup. Count indicates events/total (%). Full adjustment covariates were the same as those used in Table 2. All *p* values were two-sided, and Bold values denote statistically significant results at *p* < 0.05. Missing data were handled using complete-case analysis.

**Table 6:** Subgroup analysis using WHtR_sd.

Variable	Count	HR (95% CI)	*p* value	*P* for interaction
Overall	944/4,112 (23%)	1.18 (1.10–1.27)	**<0.001**	
Age_group				0.692
<65	540/2,195 (24.6%)	1.17 (1.07–1.28)	**<0.001**	
≥65	404/1,917 (21.1%)	1.19 (1.06–1.33)	**0.003**	
Sex				0.213
Female	370/2,048 (18.1%)	1.24 (1.10–1.40)	**<0.001**	
Male	574/2,064 (27.8%)	1.15 (1.06–1.26)	**0.001**	
Ethnicity				0.531
White	932/4,065 (22.9%)	1.18 (1.10–1.26)	**<0.001**	
Other	12/47 (25.5%)	1.00 (0.62–1.62)	0.991	
Hypertension				**0.041**
No	584/2,620 (22.3%)	1.12 (1.02–1.22)	**0.016**	
Yes	360/1,492 (24.1%)	1.27 (1.14–1.41)	**<0.001**	
Diabetes				0.588
No	888/3,840 (23.1%)	1.18 (1.10–1.27)	**<0.001**	
Yes	56/272 (20.6%)	1.16 (0.82–1.66)	0.401	
Smoking status				0.325
Current smokers	116/575 (20.2%)	1.07 (0.88–1.30)	0.520	
Ever smokers	457/1,977 (23.1%)	1.26 (1.14–1.39)	**<0.001**	
Never smokers	371/1,560 (23.8%)	1.15 (1.03–1.29)	**0.011**	

HR, hazard ratio; CI, confidence interval; SD, standard deviation; WHtR, waist-to-height ratio; P for interaction, *p* value for interaction from likelihood ratio test (models with vs. without interaction term).

HRs per 1-SD increase were estimated using fully adjusted Cox models, stratified by subgroup. Count indicates events/total (%). Full adjustment covariates were the same as those used in Table 2. All *p* values were two-sided, and Bold values denote statistically significant results at *p* < 0.05. Missing data were handled using complete-case analysis.

**Table 7:** Subgroup analysis using WC_sd.

Variable	Count	HR (95% CI)	*p* value	*P* for interaction
Overall	944/4,112 (23%)	1.21 (1.13–1.31)	**<0.001**	
Age_group				0.691
<65	540/2,195 (24.6%)	1.19 (1.08–1.31)	**<0.001**	
≥65	404/1,917 (21.1%)	1.22 (1.08–1.39)	**0.002**	
Sex				0.492
Female	370/2,048 (18.1%)	1.24 (1.09–1.40)	**0.001**	
Male	574/2,064 (27.8%)	1.20 (1.09–1.33)	**<0.001**	
Ethnicity				0.610
White	932/4,065 (22.9%)	1.21 (1.12–1.31)	**<0.001**	
Other	12/47 (25.5%)	1.47 (0.89–2.41)	0.129	
Hypertension				**0.013**
No	584/2,620 (22.3%)	1.14 (1.03–1.26)	**0.014**	
Yes	360/1,492 (24.1%)	1.32 (1.18–1.48)	**<0.001**	
Diabetes				0.736
No	888/3,840 (23.1%)	1.22 (1.12–1.31)	**<0.001**	
Yes	56/272 (20.6%)	1.21 (0.84–1.75)	0.297	
Smoking status				0.194
Current smokers	116/575 (20.2%)	1.06 (0.86–1.32)	0.570	
Ever smokers	457/1,977 (23.1%)	1.29 (1.16–1.44)	**<0.001**	
Never smokers	371/1,560 (23.8%)	1.19 (1.05–1.35)	**0.005**	

HR, hazard ratio; CI, confidence interval; SD, standard deviation; WC, waist circumference; P for interaction; *p* value for interaction from likelihood ratio test (models with vs. without interaction term).

HRs per 1-SD increase were estimated using fully adjusted Cox models, stratified by subgroup. Count indicates events/total (%). Full adjustment covariates were the same as those used in Table 2. All *p* values were two-sided, and Bold values denote statistically significant results at *p* < 0.05. Missing data were handled using complete-case analysis.

**Table 8:** Subgroup analysis using BMI_sd.

Variable	Count	HR (95% CI)	*p* value	*P* for interaction
Overall	944/4,112 (23%)	1.20 (1.12–1.28)	**<0.001**	
Age_group				0.860
<65	540/2,195 (24.6%)	1.18 (1.09–1.28)	**<0.001**	
≥65	404/1,917 (21.1%)	1.20 (1.08–1.34)	**0.001**	
Sex				0.082
Female	370/2,048 (18.1%)	1.27 (1.14–1.43)	**<0.001**	
Male	574/2,064 (27.8%)	1.16 (1.07–1.26)	**<0.001**	
Race				0.440
White	932/4,065 (22.9%)	1.19 (1.12–1.27)	**<0.001**	
Other	12/47 (25.5%)	0.82 (0.56–1.20)	0.312	
Hypertension				**0.013**
No	584/2,620 (22.3%)	1.13 (1.03–1.23)	**0.008**	
Yes	360/1,492 (24.1%)	1.27 (1.16–1.40)	**<0.001**	
Diabetes				0.773
No	888/3,840 (23.1%)	1.20 (1.12–1.28)	**<0.001**	
Yes	56/272 (20.6%)	1.21 (0.87–1.67)	0.252	
Smoking status				0.307
Current smokers	116/575 (20.2%)	1.16 (0.96–1.41)	0.121	
Ever smokers	457/1,977 (23.1%)	1.27 (1.16–1.39)	**<0.001**	
Never smokers	371/1,560 (23.8%)	1.14 (1.02–1.26)	**0.017**	

HR, hazard ratio; CI, confidence interval; SD, standard deviation; BMI, body mass index; P for interaction, P value for interaction from likelihood ratio test (models with vs. without interaction term).

HRs per 1-SD increase were estimated using fully adjusted Cox models, stratified by subgroup. Count indicates events/total (%). Full adjustment covariates were the same as those used in Table 2. All *p* values were two-sided, and Bold values denote statistically significant results at *p* < 0.05. Missing data were handled using complete-case analysis.

## Discussion

This prospective analysis involving 4,112 arthritis-free older English adults from the ELSA cohort demonstrates that several anthropometric indices reflecting central and visceral adiposity were associated with increased risk of incident self-reported physician-diagnosed arthritis over a median follow-up of 14.2 years.

In models adjusted for age, sex, ethnicity, education, marital status, smoking status, drinking status, hypertension, diabetes, stroke, high cholesterol, congestive heart failure, psychological problems, CASP-19, and moderate-to-vigorous physical activity, per-1-SD increments in BRI, WHtR, WC, BMI, and WWI were associated with a higher incidence of incident arthritis, with consistent dose–response gradients observed across tertiles and confirmed by RCS models showing predominantly linear positive relationships. In contrast, ABSI showed no statistically significant association after adjustment.

These findings extend previous cross-sectional evidence by providing prospective confirmation that several measures of visceral and central adiposity were associated with incident arthritis among older adults.

Meta-analyses have reported a 20–35% increased arthritis risk per 5 kg/m^2^ increment in BMI, with the strongest associations observed for knee joints because of mechanical loading (4, 6). However, VAT secretes pro-inflammatory adipokines (leptin, resistin, and visfatin) and cytokines (IL-6 and TNF-α) that promote synovitis and cartilage degradation even in non-weight-bearing joints (3, 7).

Some studies suggest that novel indices such as BRI and WHtR correlate with MRI-quantified VAT and may therefore better capture this inflammatory pathway (8, 11). Recent NHANES cross-sectional studies similarly reported positive associations between BRI and prevalent arthritis, with odds ratios per SD ranging from 1.15 to 1.52, broadly consistent with the longitudinal HRs observed in the present study (1.19–1.20) (12, 15).

As an observational study, the current analysis provides supportive prospective evidence but cannot establish causality.

The null finding for ABSI merits further discussion. ABSI was developed to predict mortality independent of BMI by emphasizing abdominal shape (9). In older adults, where sarcopenia often coexists with central fat redistribution, ABSI may inadvertently adjust away the very risk conferred by absolute WC (13). Previous studies in hypertension or metabolic cohorts have yielded mixed findings regarding ABSI and musculoskeletal outcomes (13).

Subgroup analyses demonstrated generally consistent associations, with evidence of effect modification by hypertension for WHtR, WC, and BMI (*P* for interaction < 0.05). Potential shared biological pathways between central obesity and hypertension warrant further investigation. No significant effect modification was observed for diabetes or smoking status. The lack of sex interaction may be related to the composite arthritis outcome and the characteristics of the study population.

Strengths of this study include its prospective design, large sample size, long follow-up duration, objectively measured anthropometrics (thereby reducing recall bias), and direct comparison of six indices within the same cohort, addressing a key gap in the literature (16).

The use of standardized z-scores facilitates clinical interpretation. ELSA’s nationally representative sampling in England enhances the generalizability of the findings to community-dwelling older adults in this setting.

Although data from other UK nations were not available because of access restrictions, ELSA provided the necessary high-quality anthropometric and longitudinal arthritis data for this analysis.

From a nutritional perspective, these results suggest the potential value of incorporating waist-based indices in addition to BMI for risk assessment in older adults. BRI and WHtR, which can be easily calculated using routine WC and height measurements, may be considered in geriatric health evaluation (11).

Lifestyle interventions aimed at reducing central adiposity (e.g., healthy dietary patterns and physical activity) may help lower arthritis risk, although direct dietary data were not available in this study.

Future longitudinal studies should incorporate joint-specific radiographic outcomes, serial inflammatory biomarkers (e.g., CRP and IL-6), and mediation analyses to quantify the contribution of VAT-derived inflammation versus mechanical loading. Well-designed randomized trials would also be valuable for evaluating whether reducing central adiposity through lifestyle interventions can lower arthritis incidence. Studies in multi-ethnic cohorts and younger populations are also warranted to explore life-course trajectories.

Several limitations should be acknowledged. Arthritis ascertainment relied on self-report. The specific ELSA question asked whether a doctor had ever told the participant that they had “arthritis (including osteoarthritis or rheumatism).”

While this question has reasonable validity for overall arthritis in older adults, it does not distinguish between osteoarthritis (the predominant form) and inflammatory arthritides such as rheumatoid arthritis, nor does it specify affected joint sites (e.g., knee, hip, or hand).

Biomechanical effects of adiposity are likely stronger for knee arthritis, whereas metabolic and inflammatory pathways may play a larger role in hand arthritis or rheumatoid arthritis. Pooling all arthritis types may therefore obscure important heterogeneity and introduce some misclassification bias.

Sensitivity analyses by arthritis subtype were not possible because of the lack of detailed diagnostic information in ELSA. Future studies with radiographic confirmation or clinical adjudication are warranted to address this limitation.

Because arthritis was ascertained at discrete survey waves, event timing is interval-censored. Events were assigned to the wave of first self-report, which is a common approach but may introduce minor bias.

Attrition and competing mortality among the oldest participants may have biased HRs toward the null, although standard Cox censoring was used. Detailed mortality data were available only at Wave 9, limiting the ability to conduct formal competing-risk analyses (Fine–Gray models).

Given the relatively low mortality rate in the analytic sample during follow-up, the impact on the main findings is likely modest; however, bias from competing events among participants with higher adiposity cannot be completely excluded.

Residual confounding by unmeasured factors, such as detailed dietary patterns, occupational joint loading, or previous joint injury, remains possible despite adjustment for a broad range of covariates.

ELSA’s nationally representative sampling enhances generalizability to community-dwelling older adults in England. However, the study population was 98.9% White, which limits the generalizability of the findings to more ethnically diverse populations. Replication in multi-ethnic cohorts is warranted to determine whether these associations are consistent across different racial and ethnic groups.

Finally, although RCS analyses suggested linearity, threshold effects at extreme values cannot be ruled out.

## Conclusion

Higher levels of BRI, WHtR, WC, BMI, and WWI at baseline were associated with an increased risk of developing arthritis over 14.2 years in a large elderly cohort, whereas ABSI showed no significant association with incident arthritis.

These results suggest the value of central adiposity markers for arthritis risk assessment in this population and support the potential benefit of lifestyle interventions aimed at reducing visceral adiposity.

## Data Availability

The original contributions presented in the study are included in the article/Supplementary material, further inquiries can be directed to the corresponding author.
