# Supplementary material for: Association between six anthropometric indices and incident arthritis: prospective findings from the English Longitudinal Study of Ageing
Source: Front Nutr. 2026 May 25;13:1824752. doi: 10.3389/fnut.2026.1824752 (PMC13243096; doi:10.3389/fnut.2026.1824752)
Supplement: Supplementary file 1 [file Table_1.DOCX]

Table S1. Baseline Characteristics by Tertiles of Body Roundness Index (BRI_sd)

|  | Low | Medium | High | *P-value* |
| --- | --- | --- | --- | --- |
| Number | 1,371 | 1,371 | 1,370 |  |
| Age, year (mean (SD)) | 63.85 (8.80) | 65.06 (8.95) | 65.79 (8.99) | **<0.001** |
| Sex (%) |  |  |  | **<0.001** |
| Female | 485 (35.4) | 769 (56.1) | 794 (58.0) |  |
| Male | 886 (64.6) | 602 (43.9) | 576 (42.0) |  |
| Race (%) |  |  |  | 0.268 |
| White | 1360 (99.2) | 1355 (98.8) | 1350 (98.5) |  |
| others | 11 (0.8) | 16 (1.2) | 20 (1.5) |  |
| Education (%) |  |  |  | **<0.001** |
| Below high school | 414 (30.2) | 487 (35.5) | 589 (43.0) |  |
| College or above | 534 (38.9) | 514 (37.5) | 434 (31.7) |  |
| High school | 283 (20.6) | 264 (19.3) | 236 (17.2) |  |
| Other | 140 (10.2) | 106 (7.7) | 111 (8.1) |  |
| Marital status (%) |  |  |  | **0.006** |
| Married or partnered | 977 (71.3) | 1054 (76.9) | 1038 (75.8) |  |
| Never married | 71 (5.2) | 46 (3.4) | 56 (4.1) |  |
| Separated/divorced/Widowed | 323 (23.6) | 271 (19.8) | 276 (20.1) |  |
| Smoking status (%) |  |  |  | **<0.001** |
| current smokers | 207 (15.1) | 196 (14.3) | 172 (12.6) |  |
| ever smokers | 572 (41.7) | 644 (47.0) | 761 (55.5) |  |
| never smokers | 592 (43.2) | 531 (38.7) | 437 (31.9) |  |
| Drinking status (%) |  |  |  | **0.018** |
| Ever drinkers | 1263 (92.1) | 1276 (93.1) | 1235 (90.1) |  |
| Never drinkers | 108 (7.9) | 95 (6.9) | 135 (9.9) |  |
| Hypertension, n (%) |  |  |  | **<0.001** |
| No | 1029 (75.1) | 898 (65.5) | 693 (50.6) |  |
| Yes | 342 (24.9) | 473 (34.5) | 677 (49.4) |  |
| Diabetes, n (%) |  |  |  | **<0.001** |
| No | 1338 (97.6) | 1293 (94.3) | 1209 (88.2) |  |
| Yes | 33 (2.4) | 78 (5.7) | 161 (11.8) |  |
| Psych problems, n (%) |  |  |  | 0.154 |
| No | 1259 (91.8) | 1282 (93.5) | 1280 (93.4) |  |
| Yes | 112 (8.2) | 89 (6.5) | 90 (6.6) |  |
| Stroke, n (%) |  |  |  | **0.006** |
| No | 1340 (97.7) | 1335 (97.4) | 1312 (95.8) |  |
| Yes | 31 (2.3) | 36 (2.6) | 58 (4.2) |  |
| High cholesterol, n (%) |  |  |  | **<0.001** |
| No | 1180 (86.1) | 1158 (84.5) | 1094 (79.9) |  |
| Yes | 191 (13.9) | 213 (15.5) | 275 (20.1) |  |
| Congestive heart failure, n (%) |  |  |  | 0.245 |
| No | 1367 (99.7) | 1368 (99.8) | 1362 (99.4) |  |
| Yes | 4 (0.3) | 3 (0.2) | 8 (0.6) |  |
| CASP-19 (mean (SD)) | 45.15 (7.76) | 44.68 (7.56) | 43.41 (8.17) | **<0.001** |
| Moderate-to-vigorous physical activity |  |  |  | **<0.001** |
| No | 993 (72.4) | 1046 (76.3) | 1161 (84.7) |  |
| Yes | 378 (27.6) | 325 (23.7) | 209 (15.3) |  |

Table S2. Baseline Characteristics by Tertiles of Waist Circumference (WC_sd)

|  | Low | Medium | High | *P-value* |
| --- | --- | --- | --- | --- |
| Number | 1,371 | 1,371 | 1,370 |  |
| Age,year (mean (SD)) | 64.84 (9.36) | 64.98 (8.96) | 64.88 (8.51) | 0.910 |
| Sex (%) |  |  |  | **<0.001** |
| Female | 273 (19.9) | 750 (54.7) | 1025 (74.8) |  |
| Male | 1098 (80.1) | 621 (45.3) | 345 (25.2) |  |
| Race (%) |  |  |  | 0.451 |
| White | 1359 (99.1) | 1352 (98.6) | 1354 (98.8) |  |
| others | 12 (0.9) | 19 (1.4) | 16 (1.2) |  |
| Education (%) |  |  |  | **<0.001** |
| Below high school | 480 (35.0) | 482 (35.2) | 528 (38.5) |  |
| College or above | 459 (33.5) | 502 (36.6) | 521 (38.0) |  |
| High school | 279 (20.4) | 268 (19.5) | 236 (17.2) |  |
| Other | 153 (11.2) | 119 (8.7) | 85 (6.2) |  |
| Marital status (%) |  |  |  | **<0.001** |
| Married or partnered | 933 (68.1) | 1052 (76.7) | 1084 (79.1) |  |
| Never married | 63 (4.6) | 52 (3.8) | 58 (4.2) |  |
| Separated/divorced/Widowed | 375 (27.4) | 267 (19.5) | 228 (16.6) |  |
| Smoking status (%) |  |  |  | **<0.001** |
| current smokers | 203 (14.8) | 193 (14.1) | 179 (13.1) |  |
| ever smokers | 546 (39.8) | 639 (46.6) | 792 (57.8) |  |
| never smokers | 622 (45.4) | 539 (39.3) | 399 (29.1) |  |
| Drinking status (%) |  |  |  | 0.068 |
| Ever drinkers | 1245 (90.8) | 1277 (93.1) | 1252 (91.4) |  |
| Never drinkers | 126 (9.2) | 94 (6.9) | 118 (8.6) |  |
| Hypertension, n (%) |  |  |  | **<0.001** |
| No | 998 (72.8) | 902 (65.8) | 720 (52.6) |  |
| Yes | 373 (27.2) | 469 (34.2) | 650 (47.4) |  |
| Diabetes, n (%) |  |  |  | **<0.001** |
| No | 1344 (98.0) | 1292 (94.2) | 1204 (87.9) |  |
| Yes | 27 (2.0) | 79 (5.8) | 166 (12.1) |  |
| Psych problems, n (%) |  |  |  | 0.290 |
| No | 1262 (92.0) | 1278 (93.2) | 1281 (93.5) |  |
| Yes | 109 (8.0) | 93 (6.8) | 89 (6.5) |  |
| Stroke, n (%) |  |  |  | 0.050 |
| No | 1338 (97.6) | 1333 (97.2) | 1316 (96.1) |  |
| Yes | 33 (2.4) | 38 (2.8) | 54 (3.9) |  |
| High cholesterol, n (%) |  |  |  | **0.006** |
| No | 1177 (85.8) | 1142 (83.3) | 1113 (81.3) |  |
| Yes | 194 (14.2) | 229 (16.7) | 256 (18.7) |  |
| Congestive heart failure, n (%) |  |  |  | 0.090 |
| No | 1368 (99.8) | 1368 (99.8) | 1361 (99.3) |  |
| Yes | 3 (0.2) | 3 (0.2) | 9 (0.7) |  |
| CASP-19 (mean (SD)) | 44.98 (7.85) | 44.69 (7.52) | 43.59 (8.15) | **<0.001** |
| Moderate-to-vigorous physical activity |  |  |  | **<0.001** |
| No | 1018 (74.3) | 1052 (76.7) | 1130 (82.5) |  |
| Yes | 353 (25.7) | 319 (23.3) | 240 (17.5) |  |

Table S3. Baseline Characteristics by Tertiles of Weight-adjusted Waist Index (WWI_sd)

|  | Low | Medium | High | *P-value* |
| --- | --- | --- | --- | --- |
| Number | 1,371 | 1,371 | 1,370 |  |
| Age, year (mean (SD)) | 62.27 (7.87) | 64.59 (8.64) | 67.85 (9.38) | **<0.001** |
| Sex (%) |  |  |  | **<0.001** |
| Female | 408 (29.8) | 756 (55.1) | 884 (64.5) |  |
| Male | 963 (70.2) | 615 (44.9) | 486 (35.5) |  |
| Race (%) |  |  |  | 0.582 |
| White | 1357 (99.0) | 1357 (99.0) | 1351 (98.6) |  |
| others | 14 (1.0) | 14 (1.0) | 19 (1.4) |  |
| Education (%) |  |  |  | **<0.001** |
| Below high school | 362 (26.4) | 501 (36.5) | 627 (45.8) |  |
| College or above | 561 (40.9) | 508 (37.1) | 413 (30.1) |  |
| High school | 312 (22.8) | 243 (17.7) | 228 (16.6) |  |
| Other | 136 (9.9) | 119 (8.7) | 102 (7.4) |  |
| Marital status (%) |  |  |  | 0.194 |
| Married or partnered | 1029 (75.1) | 1045 (76.2) | 995 (72.6) |  |
| Never married | 51 (3.7) | 59 (4.3) | 63 (4.6) |  |
| Separated/divorced/Widowed | 291 (21.2) | 267 (19.5) | 312 (22.8) |  |
| Smoking status (%) |  |  |  | **<0.001** |
| current smokers | 174 (12.7) | 179 (13.1) | 222 (16.2) |  |
| ever smokers | 564 (41.1) | 677 (49.4) | 736 (53.7) |  |
| never smokers | 633 (46.2) | 515 (37.6) | 412 (30.1) |  |
| Drinking status (%) |  |  |  | 0.127 |
| Ever drinkers | 1263 (92.1) | 1270 (92.6) | 1241 (90.6) |  |
| Never drinkers | 108 (7.9) | 101 (7.4) | 129 (9.4) |  |
| Hypertension, n (%) |  |  |  | **<0.001** |
| No | 1014 (74.0) | 872 (63.6) | 734 (53.6) |  |
| Yes | 357 (26.0) | 499 (36.4) | 636 (46.4) |  |
| Diabetes, n (%) |  |  |  | **<0.001** |
| No | 1334 (97.3) | 1294 (94.4) | 1212 (88.5) |  |
| Yes | 37 (2.7) | 77 (5.6) | 158 (11.5) |  |
| Psych problems, n (%) |  |  |  | 0.052 |
| No | 1260 (91.9) | 1270 (92.6) | 1291 (94.2) |  |
| Yes | 111 (8.1) | 101 (7.4) | 79 (5.8) |  |
| Stroke, n (%) |  |  |  | **<0.001** |
| No | 1347 (98.2) | 1332 (97.2) | 1308 (95.5) |  |
| Yes | 24 (1.8) | 39 (2.8) | 62 (4.5) |  |
| High cholesterol, n (%) |  |  |  | **0.001** |
| No | 1165 (85.0) | 1165 (85.0) | 1102 (80.5) |  |
| Yes | 206 (15.0) | 206 (15.0) | 267 (19.5) |  |
| Congestive heart failure, n (%) |  |  |  | 0.073 |
| No | 1369 (99.9) | 1367 (99.7) | 1361 (99.3) |  |
| Yes | 2 (0.1) | 4 (0.3) | 9 (0.7) |  |
| CASP-19 (mean (SD)) | 45.59 (7.39) | 44.69 (7.54) | 42.91 (8.42) | **<0.001** |
| Moderate-to-vigorous physical activity |  |  |  | **<0.001** |
| No | 977 (71.3) | 1061 (77.4) | 1162 (84.8) |  |
| Yes | 394 (28.7) | 310 (22.6) | 208 (15.2) |  |

Table S4. Baseline Characteristics by Tertiles of Waist-to-Height Ratio (WHtR_sd)

|  | Low | Medium | High | *P-value* |
| --- | --- | --- | --- | --- |
| Number | 1,371 | 1,371 | 1,370 |  |
| Age, year (mean (SD)) | 63.85 (8.80) | 65.06 (8.95) | 65.79 (8.99) | **<0.001** |
| Sex (%) |  |  |  | **<0.001** |
| Female | 485 (35.4) | 769 (56.1) | 794 (58.0) |  |
| Male | 886 (64.6) | 602 (43.9) | 576 (42.0) |  |
| Race (%) |  |  |  | 0.268 |
| White | 1360 (99.2) | 1355 (98.8) | 1350 (98.5) |  |
| others | 11 (0.8) | 16 (1.2) | 20 (1.5) |  |
| Education (%) |  |  |  | **<0.001** |
| Below high school | 414 (30.2) | 487 (35.5) | 589 (43.0) |  |
| College or above | 534 (38.9) | 514 (37.5) | 434 (31.7) |  |
| High school | 283 (20.6) | 264 (19.3) | 236 (17.2) |  |
| Other | 140 (10.2) | 106 (7.7) | 111 (8.1) |  |
| Marital status (%) |  |  |  | **0.006** |
| Married or partnered | 977 (71.3) | 1054 (76.9) | 1038 (75.8) |  |
| Never married | 71 (5.2) | 46 (3.4) | 56 (4.1) |  |
| Separated/divorced/Widowed | 323 (23.6) | 271 (19.8) | 276 (20.1) |  |
| Smoking status (%) |  |  |  | **<0.001** |
| current smokers | 207 (15.1) | 196 (14.3) | 172 (12.6) |  |
| ever smokers | 572 (41.7) | 644 (47.0) | 761 (55.5) |  |
| never smokers | 592 (43.2) | 531 (38.7) | 437 (31.9) |  |
| Drinking status (%) |  |  |  | **0.018** |
| Ever drinkers | 1263 (92.1) | 1276 (93.1) | 1235 (90.1) |  |
| Never drinkers | 108 (7.9) | 95 (6.9) | 135 (9.9) |  |
| Hypertension, n (%) |  |  |  | **<0.001** |
| No | 1029 (75.1) | 898 (65.5) | 693 (50.6) |  |
| Yes | 342 (24.9) | 473 (34.5) | 677 (49.4) |  |
| Diabetes, n (%) |  |  |  | **<0.001** |
| No | 1338 (97.6) | 1293 (94.3) | 1209 (88.2) |  |
| Yes | 33 (2.4) | 78 (5.7) | 161 (11.8) |  |
| Psych problems, n (%) |  |  |  | 0.154 |
| No | 1259 (91.8) | 1282 (93.5) | 1280 (93.4) |  |
| Yes | 112 (8.2) | 89 (6.5) | 90 (6.6) |  |
| Stroke, n (%) |  |  |  | **0.006** |
| No | 1340 (97.7) | 1335 (97.4) | 1312 (95.8) |  |
| Yes | 31 (2.3) | 36 (2.6) | 58 (4.2) |  |
| High cholesterol, n (%) |  |  |  | **<0.001** |
| No | 1180 (86.1) | 1158 (84.5) | 1094 (79.9) |  |
| Yes | 191 (13.9) | 213 (15.5) | 275 (20.1) |  |
| Congestive heart failure, n (%) |  |  |  | 0.245 |
| No | 1367 (99.7) | 1368 (99.8) | 1362 (99.4) |  |
| Yes | 4 (0.3) | 3 (0.2) | 8 (0.6) |  |
| CASP-19 (mean (SD)) | 45.15 (7.76) | 44.68 (7.56) | 43.41 (8.17) | **<0.001** |
| Moderate-to-vigorous physical activity |  |  |  | **<0.001** |
| No | 993 (72.4) | 1046 (76.3) | 1161 (84.7) |  |
| Yes | 378 (27.6) | 325 (23.7) | 209 (15.3) |  |

Table S5. Baseline Characteristics by Tertiles of Body Mass Index (BMI_sd)

|  | Low | Medium | High | *P-value* |
| --- | --- | --- | --- | --- |
| Number | 1,371 | 1,371 | 1,370 |  |
| Age, year (mean (SD)) | 65.79 (9.73) | 64.73 (8.54) | 64.18 (8.44) | **<0.001** |
| Sex (%) |  |  |  | **<0.001** |
| Female | 590 (43.0) | 780 (56.9) | 678 (49.5) |  |
| Male | 781 (57.0) | 591 (43.1) | 692 (50.5) |  |
| Race (%) |  |  |  | 0.860 |
| White | 1354 (98.8) | 1357 (99.0) | 1354 (98.8) |  |
| others | 17 (1.2) | 14 (1.0) | 16 (1.2) |  |
| Education (%) |  |  |  | **0.031** |
| Below high school | 466 (34.0) | 481 (35.1) | 543 (39.6) |  |
| College or above | 517 (37.7) | 518 (37.8) | 447 (32.6) |  |
| High school | 269 (19.6) | 255 (18.6) | 259 (18.9) |  |
| Other | 119 (8.7) | 117 (8.5) | 121 (8.8) |  |
| Marital status (%) |  |  |  | **<0.001** |
| Married or partnered | 952 (69.4) | 1060 (77.3) | 1057 (77.2) |  |
| Never married | 78 (5.7) | 47 (3.4) | 48 (3.5) |  |
| Separated/divorced/Widowed | 341 (24.9) | 264 (19.3) | 265 (19.3) |  |
| Smoking status (%) |  |  |  | **<0.001** |
| current smokers | 245 (17.9) | 166 (12.1) | 164 (12.0) |  |
| ever smokers | 590 (43.0) | 675 (49.2) | 712 (52.0) |  |
| never smokers | 536 (39.1) | 530 (38.7) | 494 (36.1) |  |
| Drinking status (%) |  |  |  | **0.002** |
| Ever drinkers | 1252 (91.3) | 1286 (93.8) | 1236 (90.2) |  |
| Never drinkers | 119 (8.7) | 85 (6.2) | 134 (9.8) |  |
| Hypertension, n (%) |  |  |  | **<0.001** |
| No | 996 (72.6) | 908 (66.2) | 716 (52.3) |  |
| Yes | 375 (27.4) | 463 (33.8) | 654 (47.7) |  |
| Diabetes, n (%) |  |  |  | **<0.001** |
| No | 1321 (96.4) | 1293 (94.3) | 1226 (89.5) |  |
| Yes | 50 (3.6) | 78 (5.7) | 144 (10.5) |  |
| Psych problems, n (%) |  |  |  | 0.475 |
| No | 1267 (92.4) | 1283 (93.6) | 1271 (92.8) |  |
| Yes | 104 (7.6) | 88 (6.4) | 99 (7.2) |  |
| Stroke, n (%) |  |  |  | 0.117 |
| No | 1332 (97.2) | 1337 (97.5) | 1318 (96.2) |  |
| Yes | 39 (2.8) | 34 (2.5) | 52 (3.8) |  |
| High cholesterol, n (%) |  |  |  | **0.001** |
| No | 1181 (86.1) | 1145 (83.5) | 1106 (80.8) |  |
| Yes | 190 (13.9) | 226 (16.5) | 263 (19.2) |  |
| Congestive heart failure, n (%) |  |  |  | **0.022** |
| No | 1363 (99.4) | 1371 (100.0) | 1363 (99.5) |  |
| Yes | 8 (0.6) | 0 (0.0) | 7 (0.5) |  |
| CASP-19 (mean (SD)) | 44.44 (7.93) | 44.91 (7.48) | 43.93 (8.14) | **0.008** |
| Moderate-to-vigorous physical activity |  |  |  | **<0.001** |
| No | 1016 (74.1) | 1050 ( 76.6) | 1134 (82.8) |  |
| Yes | 355 (25.9) | 321 ( 23.4) | 236 (17.2) |  |

Table S6. Baseline Characteristics by Tertiles of A Body Shape Index (ABSI_sd)

|  | Low | Medium | High | *P-value* |
| --- | --- | --- | --- | --- |
| Number | 1,371 | 1,371 | 1,370 |  |
| Age, year (mean (SD)) | 63.04 (8.30) | 64.26 (8.69) | 67.41 (9.26) | **<0.001** |
| Sex (%) |  |  |  | **<0.001** |
| Female | 164 (12.0) | 748 (54.6) | 1136 (82.9) |  |
| Male | 1207 (88.0) | 623 (45.4) | 234 (17.1) |  |
| Race (%) |  |  |  | 0.450 |
| White | 1355 (98.8) | 1359 (99.1) | 1351 (98.6) |  |
| others | 16 (1.2) | 12 (0.9) | 19 (1.4) |  |
| Education (%) |  |  |  | **<0.001** |
| Below high school | 444 (32.4) | 499 (36.4) | 547 (39.9) |  |
| College or above | 457 (33.3) | 521 (38.0) | 504 (36.8) |  |
| High school | 305 (22.2) | 252 (18.4) | 226 (16.5) |  |
| Other | 165 (12.0) | 99 (7.2) | 93 (6.8) |  |
| Marital status (%) |  |  |  | **<0.001** |
| Married or partnered | 988 (72.1) | 1047 (76.4) | 1034 (75.5) |  |
| Never married | 40 (2.9) | 63 (4.6) | 70 (5.1) |  |
| Separated/divorced/Widowed | 343 (25.0) | 261 (19.0) | 266 (19.4) |  |
| Smoking status (%) |  |  |  | **<0.001** |
| current smokers | 170 (12.4) | 174 (12.7) | 231 (16.9) |  |
| ever smokers | 531 (38.7) | 671 (48.9) | 775 (56.6) |  |
| never smokers | 670 (48.9) | 526 (38.4) | 364 (26.6) |  |
| Drinking status (%) |  |  |  | **0.001** |
| Ever drinkers | 1229 (89.6) | 1280 (93.4) | 1265 (92.3) |  |
| Never drinkers | 142 (10.4) | 91 (6.6) | 105 (7.7) |  |
| Hypertension, n (%) |  |  |  | **<0.001** |
| No | 946 (69.0) | 878 (64.0) | 796 (58.1) |  |
| Yes | 425 (31.0) | 493 (36.0) | 574 (41.9) |  |
| Diabetes, n (%) |  |  |  | **<0.001** |
| No | 1333 (97.2) | 1270 (92.6) | 1237 (90.3) |  |
| Yes | 38 (2.8) | 101 (7.4) | 133 (9.7) |  |
| Psych problems, n (%) |  |  |  | 0.070 |
| No | 1259 (91.8) | 1273 (92.9) | 1289 (94.1) |  |
| Yes | 112 (8.2) | 98 (7.1) | 81 (5.9) |  |
| Stroke, n (%) |  |  |  | **0.001** |
| No | 1347 (98.2) | 1328 (96.9) | 1312 (95.8) |  |
| Yes | 24 (1.8) | 43 (3.1) | 58 (4.2) |  |
| High cholesterol, n (%) |  |  |  | 0.697 |
| No | 1152 (84.0) | 1146 (83.6) | 1134 (82.8) |  |
| Yes | 219 (16.0) | 225 (16.4) | 235 (17.2) |  |
| Congestive heart failure, n (%) |  |  |  | 0.073 |
| No | 1369 (99.9) | 1367 (99.7) | 1361 (99.3) |  |
| Yes | 2 (0.1) | 4 (0.3) | 9 (0.7) |  |
| CASP-19 (mean (SD)) | 45.30 (7.49) | 44.65 (7.84) | 43.29 (8.14) | **<0.001** |
| Moderate-to-vigorous physical activity |  |  |  | **<0.001** |
| No | 1048 (76.4) | 1025 (74.8) | 1127 (82.3) |  |
| Yes | 323 (23.6) | 346 (25.2) | 243 (17.7) |  |
